# Mitigating Aflatoxin Contamination in Groundnut through A Combination of Genetic Resistance and Post-Harvest Management Practices

**DOI:** 10.3390/toxins11060315

**Published:** 2019-06-03

**Authors:** Manish K. Pandey, Rakesh Kumar, Arun K. Pandey, Pooja Soni, Sunil S. Gangurde, Hari K. Sudini, Jake C. Fountain, Boshou Liao, Haile Desmae, Patrick Okori, Xiaoping Chen, Huifang Jiang, Venugopal Mendu, Hamidou Falalou, Samuel Njoroge, James Mwololo, Baozhu Guo, Weijian Zhuang, Xingjun Wang, Xuanqiang Liang, Rajeev K. Varshney

**Affiliations:** 1 International Crops Research Institute for the Semi-Arid Tropics (ICRISAT), Hyderabad 502324, India; k.rakesh@cgiar.org (R.K.); p.arunkumar@cgiar.org (A.K.P.); P.Soni@cgiar.org (P.S.); g.sunil@cgiar.org (S.S.G.); h.sudini@cgiar.org (H.K.S.); r.k.varshney@cgiar.org (R.K.V.); 2 Crop Protection and Management Research Unit, United State Department of Agriculture–Agricultural Research Service (USDA-ARS), Tifton, GA 31793, USA; jfount1@uga.edu (J.C.F.); baozhu.guo@ars.usda.gov (B.G.); 3 Department of Plant Pathology, University of Georgia, Tifton, GA 31793, USA; 4 Oil Crops Research Institute (OCRI) of Chinese Academy of Agricultural Sciences (CAAS), Wuhan 430062, China; liaoboshou@caas.cn (B.L.); peanutlab@oilcrops.cn (H.J.); 5 International Crops Research Institute for the Semi-Arid Tropics (ICRISAT), Bamako BP 320, Mali; h.desmae@cgiar.org; 6 International Crops Research Institute for the Semi-Arid Tropics (ICRISAT), Lilongwe PB 1096, Malawi; P.Okori@cgiar.org (P.O.); S.Njoroge@cgiar.org (S.N.); J.Mwololo@cgiar.org (J.M.); 7 Crops Research Institute (CRI) of Guangdong Academy of Agricultural Sciences (GAAS), Guangzhou 510640, China; chenxiaoping@gdaas.cn (X.C.); liangxuanqiang@gdaas.cn (X.L.); 8 Department of Plant and Soil Science, Texas Tech University, Lubbock, TX 79409, USA; venugopal.mendu@ttu.edu; 9 International Crops Research Institute for the Semi-Arid Tropics (ICRISAT), Niamey BP 12404, Niger; H.Falalou@cgiar.org; 10 Institute of Oil Crops, Fujian Agriculture and Forestry University, Fuzhou 350002, China; weijianz1@163.com; 11 Shandong Academy of Agricultural Sciences, Jinan 250108, China; xingjunw@hotmail.com

**Keywords:** *Aspergillus flavus*, aflatoxin contamination, groundnut, genetic resistance, post-harvest management

## Abstract

Aflatoxin is considered a “hidden poison” due to its slow and adverse effect on various biological pathways in humans, particularly among children, in whom it leads to delayed development, stunted growth, liver damage, and liver cancer. Unfortunately, the unpredictable behavior of the fungus as well as climatic conditions pose serious challenges in precise phenotyping, genetic prediction and genetic improvement, leaving the complete onus of preventing aflatoxin contamination in crops on post-harvest management. Equipping popular crop varieties with genetic resistance to aflatoxin is key to effective lowering of infection in farmer’s fields. A combination of genetic resistance for in vitro seed colonization (IVSC), pre-harvest aflatoxin contamination (PAC) and aflatoxin production together with pre- and post-harvest management may provide a sustainable solution to aflatoxin contamination. In this context, modern “omics” approaches, including next-generation genomics technologies, can provide improved and decisive information and genetic solutions. Preventing contamination will not only drastically boost the consumption and trade of the crops and products across nations/regions, but more importantly, stave off deleterious health problems among consumers across the globe.

## 1. Introduction

Aflatoxin contamination is a food safety concern that has adverse financial implications and health consequences in groundnut growing regions across the globe. Aflatoxins are a group of mycotoxins and highly toxic secondary metabolites produced by four *Aspergillus* species such as *Aspergillus flavus*, *Aspergillus parasiticus*, *Aspergillus nomius* and *Aspergillus tamarii* [1,2,3]. Groundnut, cotton, sunflower, wheat, corn and rice are key hosts for fungus causing aflatoxin outbreaks in the food chain [4]. Aflatoxins contain alternating groups of carbonyl and methylene called polyketides that are carcinogenic, immunosuppressive, hepatotoxic and teratogenic to humans and animals [5,6]. Among four prevalent groundnut aflatoxins, B1 and B2 are produced by *A. flavus* while G1 and G2 are produced by *A. parasiticus*. In addition to health issues, aflatoxin levels beyond a threshold hamper the export quality of groundnuts 20 parts per billion (ppb) in the USA and 4 ppb in Europe [7] resulting in significant economic loss to farmers and traders. Since aflatoxin decomposes at 237–306 °C, cooking, drying, pasteurization and sterilization cannot reduce aflatoxin levels in food [8]. Although calcium chloride, alkaline cooking and steeping, hydroxides and bicarbonates can reduce 84%–95% aflatoxin content [9,10,11], these treatments also reduce the nutritional quality of the product. If pre-harvest infection in crops can be avoided through host resistance, then managing post-harvest contamination becomes possible using different technologies and practices.

Inconsistent phenotyping results from aflatoxin contamination experiments suggest a large variation in germplasm due to genotype-by-environment (G×E), but studies have not led to the identification of stable resistance sources among diverse germplasm. Studies have reported the existence of three types of host-pathogen resistance mechanisms, namely in vitro seed colonization (IVSC), pre-harvest aflatoxin contamination (PAC) and aflatoxin production (AP) detected in different genetic backgrounds [12,13,14,15,16]. However, there are currently no reports on the presence of all three resistance mechanisms in a single genetic background. This provides an opportunity to combine the three resistance mechanisms to achieve stable genetic resistance against *Aspergillus* infection in the field. Speedy advancements in low-cost sequencing technologies and big data analysis provide opportunities to dissect this tricky trait by deploying complex multi-parent genetic populations (such as MAGIC—multi-parent advanced generation intercross, and NAM—nested association mapping) [17,18,19]. Further, reliable ELISA-based phenotyping of the three mechanisms on large-scale segregating breeding lines may facilitate the identification of promising lines with resistance to all the mechanisms.

Over the last decade, next-generation sequencing (NGS) technologies accelerated the development of different genomic resources in a given crop that are very affordable even for large genome-polyploid crops such as groundnut [18,19]. As a result, high quality reference genomes have been successfully developed for diploid progenitors *Arachis duranensis* [20,21] and *A. ipaensis* [21,22] wild tetraploid *A. monticola* [23] and also for both the subspecies namely, *A. hypogaea* spp. *hypogaea* [24] and *A. hypogaea* spp. *fastigiata* [25,26] of cultivated groundnut. In addition, comprehensive gene expression atlases are also available [27,28], large-scale genome-wide genetic markers and high density 58K SNPs ‘Axiom_*Arachis*’ Array [29] for use in different structural and functional genomics studies. In addition, genetic and transcriptome studies on different mechanisms provide a better understanding of biochemical pathways involved in aflatoxin biosynthesis [30,31,32]. Transgenic technology was deployed to achieve ~90% reduction in aflatoxin content in groundnut by silencing the aflatoxin producing genes (*aflR*, *aflS*, *aflep* and *aflC*) using RNAi approach [33]. Transgenic events with reduced levels of aflatoxin were recently developed in groundnut by silencing the *aflM* and *aflP* genes through host-induced gene silencing (HIGS) and overexpressing of antifungal plant defensins *MsDef1* and *MtDef4.2* [34].

**Figure 1:**
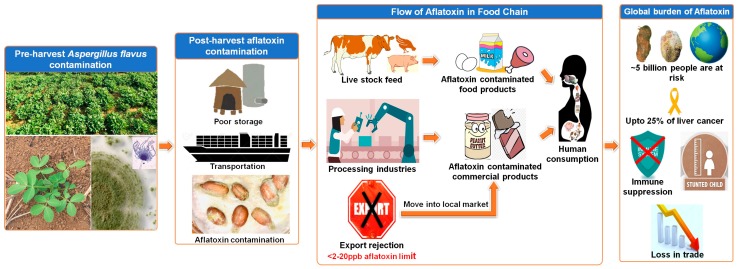
Impact of aflatoxin contamination across the groundnut value chain. Poor storage and inappropriate transportation procedures are the bottlenecks in the post-harvest stage of harvest aflatoxin contamination, subsequently causing financial loss to farmers and traders. Once these contaminated products enter the food-feed chain and travel across it, they can have an adverse impact on human health.

Since none of the above-mentioned efforts have provided heritable resistance in the genetic background of popular cultivars, serious efforts are warranted in achieving stable genetic resistance. However, it must be mentioned that post-harvest management practices have been very instrumental in mitigating contamination in the entire food chain. This paper discusses the importance and strategy of combining different resistance mechanisms together with well-tested pre- and post-harvest management and safety practices to deliver aflatoxin-free groundnuts to the food chain (Figure 1). It also advocates the deployment of modern scientific tools and technologies to minimize contamination throughout the value chain, thereby ensuring food safety and consumers’ health globally.

## 2. Characterization of Aflatoxin Producing Pathogens

Polyketide derived aflatoxins are produced by *A. flavus* which is a soil saprophytic fungus and opportunistic pathogen that impacts human and animal health. *A. flavus* grow well at 28–30 °C and 25–35 °C and is readily able to colonize in most environments whenever there is a suitable nutrient rich source of carbon and nitrogen [35] *A. flavus* mode of replication is majorly by asexual reproduction, but it also forms sclerotia or conidia in soil and in plant tissue as hardened masses of desiccated and melanized mycelia that are able to survive adverse nutritional conditions [36]. However, an earlier study reported that sexual reproduction also takes place among compatible groups of *A. flavus* strains [37]. Furthermore, colonies which are produced by *A. flavus* are powdery masses of yellow-green spores and reddish gold on the upper and lower surface area. Most of the plants which were invaded and/or colonized by *A. flavus* do not show any visible symptoms on the foliage except aflatoxin accumulation in the grains/seeds.

Groundnut pods develop beneath the soil, which is the main source of inoculum for *A. flavus* leading to infection in groundnut seeds [38,39]. The toxigenic pathogens produce a high level of toxins in the infected seeds with high protein and lipid content [40]. Based on phenotyping, toxigenic strains can be categorized into S and L type. S strains produce high levels of aflatoxins and numerous sclerotia (average diameter < 400 µm) whereas L strains produce large sclerotia but fewer aflatoxins [41] Aflatoxins (AFs) are secondary metabolites produced by the fungal plant parasite pathogens i.e., *A. flavus* and *A. parasiticus* [42]. Aflatoxins belong to a family of compounds called difuranocoumarins and are grouped under AFB_1_, AFB_2_, AFG_1_ and AFG_2_ based on fluorescence emission and their relative mobility on silica gel. Of these, AFB_1_ is most toxic and is produced by both *A. flavus* and *A. parasiticus*. AFG_1_ and AFG_2_ are produced exclusively by *A. parasiticus*. Infections from *A. flavus* are more prevalent in Asia and Africa while *A. parasiticus* is prevalent in Americas. Groundnuts tend to be colonized and contaminated by *Aspergillus* sp. at different stages and aflatoxin production occurs at pre-harvest, during harvest, post-harvest drying, in storage and also during transportation along the value chain [43,44].

Aflatoxin contamination in groundnuts is aggravated by heat and drought stresses [45,46]. Under drought conditions, aflatoxin contamination increases due to reduced moisture in the pod, resulting in cracks in the pod wall that allows the penetration of *A. flavus* [47]. Damaged pods have more aflatoxin compared to undamaged shells [48]. Under drought conditions, the production of phytoalexin is inhibited by decreasing kernel water activity, which increases aflatoxin contamination [49]. Thus, drought predisposes groundnut to aflatoxin production [50]. Although drought intensity increases aflatoxin contamination, drought tolerance does not lead to less aflatoxin contamination [51]. It has been reported that expression of AFs biosynthetic gene cluster [52] and aflatoxin production have been regulated through various environmental and nutritional factors including carbon sources [53] and oxygen availability [54,55,56,57].

The aflatoxin biosynthetic pathway has been well characterized in *A. parasiticus* and *A. flavus* [58]. Extensive research has identified a 70 kb DNA cluster consisting of two specific transcriptional regulators (aflR and aflS) and 25 co-regulated downstream metabolic genes in the aflatoxin biosynthetic pathway [59,60,61,62]. On average, about 2.8 Kb of the genomic DNA region contains one gene. The genomic region has three large gene fragments of about 5–7 Kb each for the fatty acid synthase *α* (*FAS α*), *FAS β* and the polyketide synthase (*PKS*). The average size of the other 22 genes is about 2 Kb towards 5’ end with no ORF. The expression of the two transcriptional regulators (aflR and aflS) are controlled by many regulators, i.e., CreA transcription factor, VelB/VeA/LaeA complex, and a cell surface-localized G-protein coupled receptor complex [52,63]. Most early studies focused on controlling AFB_1_ production in crops with a few dwelling on factors responsible for aflatoxin contamination.

## 3. Adverse Impacts of Aflatoxin Contamination on Human Health and the Economy

Aflatoxin adversely affects >5 billion people who are chronically exposed to a large amount (>1000 ppb) of toxin [64]. The exposure to high aflatoxin influences various biological pathways in humans through the interaction of epoxide with proteins and DNA. Exposure to the toxic effects of aflatoxin negatively affects nutrition of poor people as well as the economy, accounting for 40% of prevalent diseases affecting health [65]. Aflatoxin also has implications on the economic, social and political aspects of society. Aflatoxin is predominantly perceived as leading to aflatoxicosis and exists in two forms of acute intoxication leading to liver damage and chronic subsymptomatic exposure [65]. At present, the global burden of aflatoxin-driven hepatocellular carcinoma (HCC) or liver cancer is around 25%, mostly prevalent in developing countries due to poor post-harvest management and regular consumption of food contaminated with aflatoxin [66,67]. In the 1960s, the death of 2219 chicks in poultry farms in Mysore in Karnataka state of India, led to the origin of the word ‘aflatoxicosis’ [68]. Deaths in poultry due to aflatoxin were also reported in 1961 in turkeys fed imported (and contaminated) groundnut meal (Turkey “X” disease) [69,70]. A deleterious mutation in the P53 tumor-suppressor gene and activation of dominant oncogenes leads to hepatomas (64% of cancers) which are a predominant cancer [71,72]. Due to these implications on health, aflatoxin was placed on the list of Rapid Alert System for Food and Feed (RASFF) of the European Union in 2008. Aflatoxin (AFB_1_) has also been categorized as a class 1 carcinogen by the International Agency for Research on Cancer (IARC). This has resulted in the regulation of toxins to very low concentrations, i.e., 20 ppb in grains and 0.5 ppb in milk in the United States and 4 ppb in food for direct consumption (including groundnut) in some European countries [7].

In addition to groundnuts, aflatoxin also contaminates linseeds, sunflower seeds, cereals, beans and poultry due to contaminated feed [73]. Reports suggest that aflatoxin contamination in agricultural crops may lead to an annual loss of more than US$ 750 million in Africa [74]. In the USA, aflatoxin contamination leads to an annual income loss of more than US$ 100 million [75]. High levels were detected in children in South Africa, Durban, Nigeria and Sudan [76,77,78]. It also leads to the rejection of valuable products in the international market [79]. Products that do not meet the aflatoxin standards are rejected at the channel of distribution or sold cheaply and enter the local market [80]. In Eastern Cape area of South Africa, the most predominant aflatoxin B1 toxin was found at a concentration of 27,163 and 16,505 ppb in groundnut butter provided to children. However, 10 ppb is the maximum concentration, of which 5 ppb was that of B1 aflatoxin (http://scienceinafrica.com/health/aflatoxin-peanut-butter-mrc-policy-brief).

Levels of aflatoxin contamination depend on the *Aspergillus* species, growing and storage conditions and differ from country to country [81,82]. Factors such as genotype, soil texture, moisture deficit and insect infestation also have a bearing on severity of contamination [82]. Data on annual consumption from different countries show that exposure to aflatoxin was 11.7–2027 ng/kg/day in southern Guangxi province of China, 3.5–14.8 ng/kg/day in Kenya, 38.6–183.7 ng/kg/day in Mozambique, 11.4–158.6 ng/kg/day in Swaziland and 16.5 ng/kg/day in Transkei. In Thailand, it was 6.5–53 ng/kg/day whereas in the United States it was estimated to be 6.5–53 ng/kg/day [83]. A study in Ghana revealed estimated aflatoxin exposure in groundnut to be 9.9–99.2 ng/kg/day [84].

Standard food safety parameters set by national regulatory bodies in different countries for the benefit of human health and permissible limits differ among countries [85]. This may lead to trade loss due to high cost of meeting the standards and cost of testing, and eventual loss of admissibility into foreign markets [86]. According to food safety and standard regulations the permissible limit for aflatoxin in food commodities for sale in the Indian market is 30 μg/kg or ppb, while the tolerance value for aflatoxin M in milk is 0.5 μg/kg. However, the European Union (EU) has stringent limits for aflatoxin in dried nuts, cereals and spices, ranging from 2–12 μg/kg for B1 aflatoxin to 4–15 μg/kg for total aflatoxins, whereas, for infant foods, the range varies from 0.10 to 0.25 μg/kg [87].

## 4. Current Understanding of Resistance to Aflatoxin Contamination Based on Genetic, Genomic, Transcriptomic and Proteomic Studies

**Figure 2:**
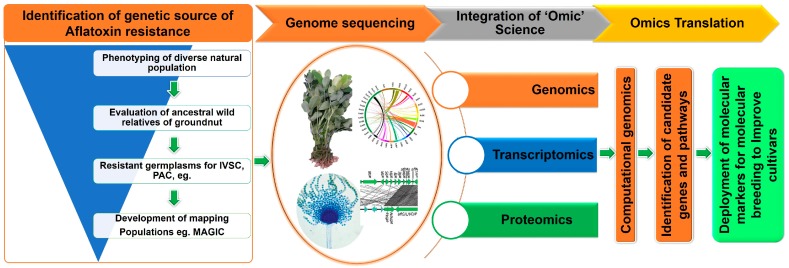
Deployment of a combination of genetic and omics approaches will develop better understanding of the pathways and genomic tools which will help in tackling aflatoxin contamination in crops through genetic improvement using genomics-assisted breeding.

Deciphering genotype to phenotype association requires an understanding of gene networks and pathways of biological systems to target complex traits such as aflatoxin contamination in groundnut. Multiple efforts have been made for phenotyping diverse groundnut genotypes leading to identification of several promising lines showing resistance/moderate resistance for *A. flavus* infection and aflatoxin production (Table 1). Modern approaches such as molecular genetics, genomics, transcriptomics and proteomics studies in groundnut have become increasingly more effective (Figure 2). A very recent study on identification of linked markers for aflatoxin resistance reported quantitative trait loci (QTLs) in a recombinant inbred line (RIL) population (Zhonghua 10 × ICG 12625) [88]. The phenotyping of this population was performed for percent seed infection index (PSII), and aflatoxin B1 (AFB_1_) and aflatoxin B2 (AFB_2_) content. Two QTLs for PSII and 12 QTLs for aflatoxin accumulation were detected by unconditional analysis. Interestingly, four QTLs (*qAFB1A07* and *qAFB1B06.1* for AFB_1_ and *qAFB2A07* and *qAFB2B06* for AFB_2_) showed major and stable effects (9.32%–21.02% PVE) [88]. It was important to note the discovery of two co-localized genomic regions on A07 (not only *qAFB1A07* and *qAFB2A07*) and on A06 (*qAFB1B06.1* and *qAFB2B06*). A closer look at the genotyping and phenotyping data suggested additive effects between two QTLs (*qAFB1A07* and *qAFB1B06.1*) leading to low AFB_1_ and AFB_2_ accumulation [88]. These are encouraging results in addition to several comprehensive trait mapping studies underway using association mapping panel and bi-parental and multi-parental genetic populations and transcriptomics at leading research organizations such as ICRISAT-India, University of Georgia-USA, USDA-USA, and Oil Crops Research Institute of the CAAS-China.

Genome sequencing of hosts and pathogens have provided insights into complex genetic architecture and novel genes. Further, transcriptomic and proteomic studies have supplemented information on gene expression and the pathways enrichment that govern phenotype. Previously, efforts were made in large-scale sequencing of cDNA to discover the gene expression pattern between susceptible and resistant genotypes and identified defense-related genes upon *A. flavus* seed colonization [97,98,99]. However, these studies were based on EST or microarray-based approaches which are less sensitive and provide low coverage for differentially expressed genes. Now, new tools such as sequencing-based RNA-seq have greatly accelerated insights into the molecular understanding of toxin production by pathogens and resistance mechanisms in different crops including groundnut [100,101,102]. This highly sensitive approach allows efficient discovery of differentially expressed genes on a larger scale. For instance, a recent RNA-seq-based approach enabled the detection of around 129,000 unigenes in groundnut seed upon *Aspergillus* infection [30,31,32]. Similarly, an RNA-seq-based transcriptomic study discovered 14,592 genes, of which 13,875 were previously annotated and 717 were novel to the *Aspergillus* spp. [101]. These studies have profound gene annotation [101] and identified key pathways responsible for mycotoxin production by *Aspergillus* sp. [102].

Proteomic studies have greatly enhanced the understanding of gene regulation and regulatory networks to explain the molecular mechanism involved in host-pathogen interaction and aflatoxin contamination [103,104,105] (Table 2). The two-dimensional difference gel electrophoresis (2-D DIGE) based proteomic approach identified a number of proteins corresponding to aflatoxin production in groundnut upon infection by toxigenic strains of *A. flavus* [99]. Though the study reported only 400 protein spots, it provided evidence of host-pathogen interaction by capturing proteins involved in DNA and RNA stabilization, biosynthesis of phytoalexins, immune response, detoxification and metabolic regulation [106]. Recently, a study deployed proteomic approach to gain insights into the pathogen regulatory mechanism involved during oxidative stress, similar to drought stress in groundnut. To reproduce oxidative stress, three isolates—AF13, NRRL3357 and K54A—with high, moderate, and no aflatoxin production, were exposed to H_2_O_2_ and their global proteome variations were studied. As a result, 1173 proteins were identified, and among them 220 were differentially expressed, controlling toxigenic abilities of strains AF13, NRRL3357 and K54A. This suggests that the toxin production abilities of toxigenic strains involve a group of genes that together regulate production of lytic enzymes, oxidative stress tolerance, production of secondary metabolites, pathogenicity, mycelial development, carbohydrate metabolism, etc. [102,106]. Together, these studies have advanced our current understanding of genetic regulations and the molecular networks involved in host-pathogen interactions to come up with new strategies in alleviating aflatoxin contamination in groundnut and other crops. For instance, this knowledge has helped researchers and breeders develop aflatoxin contamination-free transgenic maize [107] and groundnut [34] through host-induced gene silencing.

## 5. Pathways Impacting Host-Pathogen Interaction and Toxin Production

The genome of *A. flavus* translates 12,000 functional genes and comprises duplication of some lineage-specific genes which give rise to the larger genome i.e., 37 Mb [110]. The complex genome of *A. flavus* is anticipated to contain ~56 secondary metabolite gene clusters [111,112]. Among them, the aflatoxin cluster consists of aflatoxin biosynthetic genes as well as pathway-specific regulatory genes, which include 25 genes that span approximately 70 Kb of DNA [61]. Further, the aflatoxin gene cluster is positioned adjacent to the telomeric region of the third chromosome and is surrounded by four sugar-utilization genes at the distal end [113].

In *A. flavus*, the production of secondary metabolites is a highly coordinated molecular process involving a dynamic network of transcription factors that orchestrate the coordinated expression of the target biosynthetic genes of the pathogen and suppression of the host immune responses. The aflatoxin biosynthesis pathway-specific regulatory gene *aflR* encodes a DNA-binding zinc-cluster protein that binds to the promoter region of the aflatoxin pathway genes to trigger their expression [114]. Therefore, overexpression of *aflR* increases the transcript abundance of aflatoxin pathway genes [115]. Aflatoxin production is also regulated by a pathway-specific regulatory gene *aflS*, located divergently next to *aflR*, separated by a small intergenic region and having an independent promoter. Unlike *AflR*, the role of *AflS* is still not well defined because the deletion of *aflS* does not influence expression of aflatoxin biosynthetic genes. However, surprisingly, the deletion of gene *aflS* can abolish aflatoxin production in *A. flavus* [116]. Therefore, it is presumed that *AflS* can plausibly play a crucial role in aflatoxin biosynthesis through transcriptional regulation of expression of *AflR* [117], because a synergistic relationship has been observed between *AflR* and *AflS* in *A. parasiticus* [118]. More recently, a study revealed that *AflS* is essential for appropriate transportation of *AflR* to or from the nucleus and that it assists in *AflR* localization [119]. Further, DNA methylation plays an important role in mycelial development and secondary metabolism of *A. flavus*. The knock-out of *DmtA* (a putative cytosine methyltransferase) in *A. flavus* could result in reduced conidiation and sclerotial production, and attenuate strain virulence due to suppression of *aflC*, *aflK*, *aflO*, *aflS* and *aflR* [120]. Recently, a polyamine biosynthetic gene, spermidine synthase (*spds*), has been characterized as a key gene required by *Aspergillus* toxigenic strain controlling fungus seed colonization, aflatoxin production and pathogenesis [102].

Despite years of research, geneticists and molecular biologists have yet to find a stable and effective genetic solution to aflatoxin contamination. To date, breeders have identified groundnut germplasm resistant to pre-harvest and post-harvest aflatoxin contamination. Groundnut resistance to *Aspergillus* spp. involves the production of resveratrol (a natural phytoalexin) by developing seed. Resistant varieties with increased production of resveratrol upon infection exhibit enhanced resistance to in vitro seed colonization [31]. The host defense mechanism involves oxidative homeostasis in response to reactive oxygen species (ROS) formed upon *Aspergillus* infection. This is achieved by the expression of a wide range of genes involved in ROS detoxification, such as resveratrol synthase, phenylalanine ammonia lyase, chalcone synthase, catalases, superoxide dismutase, glutathione-S-transferase, senescence-associated protein, etc, [31]. Expression of these genes is important to block *Aspergillus* growth and aflatoxin production [57]. These resistance-conferring genes are involved in producing compounds such as phenylpropanoids, coumarins, stilbenes, cinnamic acid, flavonoids, and ascorbate, etc., which are the primary constituents of the groundnut seed coat [30,121]. Furthermore, transcription factors such as *WRKY*, *ERF* and *NAC* are important transcriptional regulators of antioxidant- and pathogenesis-related genes [31,32]. These genes also play an important role in the biosynthesis of volatile compounds such as jasmonate and salicylate [32], and control innate immunity [122]. The genes encoding β-1,3-glucanases, chitinases, pathogenesis-related proteins and ribosome inactivating proteins (RIPs) are key controllers of *A. flavus* resistance [57]. It is anticipated that future research will be more focused on dissecting this trait to allow researchers to develop aflatoxin-free groundnuts.

## 6. Integrated Approach for Discovering Genomic Regions and Candidate Genes

Resistance to *A. flavus* infection and aflatoxin production is one of the most complex traits influenced by several non-genetic factors such as water stress, population diversity and density of microorganisms in the soil. Nevertheless, extensive phenotyping of large scale germplasm during multiple seasons has identified several lines with minimum infection and aflatoxin production (Table 1). These resistant genotypes are currently being deployed for breeding aflatoxin-resistant lines and to develop different types of genetic populations such as association mapping panels, bi-parental and multi-parent populations [18]. Efforts are also on to deploy transcriptomics, proteomics and metabolomics approaches for a better understanding of the genes, pathways and networks involved in controlling all the three resistance mechanisms, namely IVSC, PAC and AP (see Section 5 and Table 2). ICRISAT, together with its partners, is working on such an integrated approach wherein a MAGIC population and bi-parental populations are planned to be used for genetic mapping and QTL discovery while a mini core collection will be used for association analysis to discover marker-trait associations (MTAs). These studies are likely to facilitate the identification of genomic regions controlling aflatoxin resistance. At the same time, a transcriptomic approach has been deployed to study the functional genomics of resistance mechanisms IVSC, PAC and AP by conducting separate RNAseq experiments. These integrated approaches comprising of genetics, structural genomics and functional genomics together with next-generation sequencing and comprehensive analysis will provide precise information on candidate genes to facilitate the development and validation of genetic markers for use in molecular breeding.

## 7. Moving Towards Genomics-Assisted and Transgenic-Based Genetic Improvement to Confer Aflatoxin Resistance

Developing groundnut cultivars with pre-harvest aflatoxin contamination has been one of the most challenging goals of breeding programs across the globe [123]. Conventional breeding efforts have met with very limited success in breeding aflatoxin-resistant varieties. Majority of the popularly grown varieties across the globe are susceptible to *Aspergillus* infection and aflatoxin contamination. Given the genetic complexity of this trait, GAB has the potential to enable swifter development of improved varieties with resistance to *Aspergillus* infection and aflatoxin contamination. However, discovering linked and validated markers is a pre-requisite for deploying GAB. Additionally, a low-density SNP panel (10–50 SNPs) can be developed to perform early generation selection to identify the best lines with resistance to *Aspergillus* infection and aflatoxin contamination. Limited efforts have been made so far in identifying genomic regions for PAC resistance [88,124], and, therefore, diagnostic markers are not available for use in deploying GAB. Nevertheless, several genetic mapping and association mapping studies on diverse genetic populations are underway and in coming years, multi-parent populations and cost-effective sequencing technologies together with precise phenotyping will facilitate high resolution mapping for aflatoxin resistance to develop a panel of diagnostic markers.

Several studies have reported the use of RNAi to suppress *A. flavus* growth and aflatoxin production in groundnut. For example, an hpRNA construct was successfully deployed in suppressing the expression of five genes (*aflR*, aflatoxin gene cluster transcriptional activator; *aflS*, aflatoxin gene cluster transcriptional co-activator; *aflC*, aflatoxin polyketide synthase; *aflep*, a putative aflatoxin efflux pump; and *pes1*, a NRPS responsible for tolerance to oxidative stress) involved either directly or indirectly in aflatoxin biosynthesis [33]. The transgenic groundnut lines showed up to 100% reduction in AFB_1_ and AFB_2_ compared to the control. Another study achieved high level of aflatoxin resistance by overexpressing antifungal defensins genes (*MsDef1* and *MtDef4.2*) through host-induced gene silencing (HIGS) of *aflM* and *aflP* genes from the aflatoxin biosynthetic pathway [34]. Aflatoxin B_1_ levels fell from an average of 2000 ppb in controls to less than 20 ppb (the maximum level allowed by the US FDA) in the RNAi lines as determined by highly sensitive HPLC detection methods. A strong positive correlation was observed between reduction in aflatoxin levels and aflatoxin biosynthetic gene expression using qRT-PCR. These transgenic events will be subjected to further trait verification and testing to identify the most promising events with high level of resistance to *Aspergillus* infection and aflatoxin contamination.

## 8. A Mix of Genetic Resistance, Effective Post-Harvest Management Practices and Safe Storage

*Aspergillus* infection in groundnuts is usually influenced by the aggressiveness of the fungus, genotype susceptibility, as well as soil moisture and temperature parameters [125]. Moisture stress, especially terminal drought, predisposes groundnut to *A. flavus* infection and aflatoxin contamination [50,51]. Hence aflatoxin control and prevention strategies mainly include blocking the infection process of *A. flavus* by host-plant resistance/tolerance, biological control, managing environmental factors, pre-harvest crop management and finally post-harvest crop management such as drying and storage technologies [126,127]. Under the present scenario, genetic resistance alone cannot eliminate the problem of aflatoxin contamination unless it is used in combination with other pre- and post-harvest management practices [128].

Pre-harvest management includes following in situ (in field) water management techniques which enable the crop to avoid moisture stress at critical stages. Research on the efficacy of water management techniques such as tied ridges and mulching in Zambia have proved that they were effective in significantly reducing pre-harvest aflatoxin contamination in groundnuts [129]. Other cultural practices such as the application of gypsum, a calcium amendment, proved effective in reducing aflatoxin contamination [44,130]. Application of manure has also been shown to reduce aflatoxin contamination [44,131]. Further research has confirmed that biocontrol using non-aflatoxigenic *A. flavus* and *A. parasiticus* strains significantly reduce aflatoxin contamination in groundnuts and maize [132,133].

In the case of groundnut, post-harvest handling of pods from harvest to storage is critical in managing aflatoxin build-up. High pod/seed moisture increases post-harvest molding and aflatoxin contamination. Hence proper drying of pods after harvest to 7% moisture levels is ideal to prevent the growth of fungi, including aflatoxigenic strains [134]. Earlier research confirmed that inverted windrowing after harvest avoid soil contact of pods, exposes pods to sunlight and reduces groundnut aflatoxin contamination [135]. Research on post-harvest handling showed that dried pods have lower levels of aflatoxins than pods that were not dried. The windrowing, immediate stripping and mat drying of pods are cost effective in controlling damage/molding and subsequent aflatoxin contamination [136]. Storage is another important aspect that care needs to be taken in, in the case of groundnut. Current farmer practice is to use jute and woven polypropylene bags to store groundnut [137]. Storing pods in jute bags provides conditions conducive to mold growth, especially with *A. flavus*. Jute bags are highly porous and can easily absorb moisture, and therefore foster the rapid growth and multiplication of these aflatoxigenic molds. Alternatively, hermetic storage offers a new alternative to traditional storage of grains and pods, and is a sustainable practice. Hermetic storage works on the principle of creating airtight conditions in which oxygen levels are lowered for insect, fungal and seed respiration. In a recent study conducted at ICRISAT, Purdue Improved Crop Storage (PICS) bags that rely on the principle of hermetic storage were used to safeguard groundnuts against *A. flavus* infestation, and subsequently lowered aflatoxin contamination levels in storage [138].

## 9. Challenges and Opportunities

The age-old problem of aflatoxin contamination has yet to find a sustainable and stable solution. Given its adverse impact on health, the west has set up very stringent criteria for import that prohibits many countries in Africa and Asia from selling their produce to these countries. It is also important to note that the population in the Asian and African countries has shown great tolerance to aflatoxin. Some studies have shown aflatoxin contamination in abundance in the entire food chain, with deleterious effects on the health of consumers. Since *A. flavus* infection causes no yield loss to the producer and no immediate health impact on consumers, farmers in Asia and Africa have not shown great keenness in adopting modern pre-harvest and post-harvest management practices in the processing, packaging, transportation and storage stages. Many farmers are not even aware of the adverse health impact.

Given that health and commerce are equally important, it is essential to minimize aflatoxin contamination in the entire food chain. This would entail deploying more precise phenotyping and diverse genetic populations together with different “omics” approaches to identify genomic regions and candidate genes for accelerated breeding through GAB. Genetic resistance will provide the much needed defense from infection in the field and post-harvest management will ensure less aflatoxin in the produce. A combination of recent advancements in modern genetics, genomics, phenomics resources, tools and technologies along with pre-post-harvest management practices could potentially provide a stable and long term solution for this complex problem.

## Figures and Tables

**Table 1:** A summary of the screening and characterization of groundnut germplasm using different phenotyping methods leading to identification of aflatoxin-resistant genotypes.

Resistance Mechanism	Sample Size and Material Type	Toxigenic Species	Screening Method	Significant Outcome	Resistant Varieties Identified	Reference
**1. PHAC**	13 elite varieties and landraces	*A. flavus*	Mycelial growth on the surface of kernels	The compact arrangement of palisade-like layers of the seed testa is resistant to PHAC	Igola, Serenut 1, Serenut 2 and entry 99527	[14]
	10 elite varieties	*A. flavus*	Seed infection coverage and intensity analyzer (SICIA)	All groundnut genotypes support PHAC, but AP varies among genotypes	ICG 1471, NC3033, ICGV 88145, GT-C20	[15]
**2. PAC**	7 elite varieties	*A. flavus*	Green conidial heads of *A. flavus* group and black conidial heads of *A. niger*	Low levels of linoleic acid do not affect aflatoxin production during PAC	F1334 and F1344	[89]
	11 germplasm lines	*A. flavus*	Aflatoxin estimated using ELISA	PAC increases when exposed to terminal drought	ICGV 98305, ICGV 98348, ICGV 98353, Tifton 8	[47]
**3. IVSC**	40 elite varieties	*A. flavus*	Seeds with *A. flavus* colonies were counted	IVSC increases with increased drought stress	55–437, PI 337409, PI 337394F, 73–30	[90]
	35 wild accessions	*A. flavus*	IVSC and AP	*A. pusilla*, *A. chiquitana*, *A. triseminata* species resistant to IVSC and AP	ICG 13212, ICG 11560, ICG 8131, ICG 14875	[13]
	37 cultivars	*A. flavus*	*A. flavus* inoculated	Different varieties produce aflatoxin B1 and B2 at different levels during IVSC	PI 337394F, PI 337409, J-11	[91]
	>100 accessions, breeding lines and commercial varieties	*A. flavus*	Visual development of conidial spores	Lower moisture has higher level of resistance to penetration by *A. flavus* during IVSC	PI 337394, PI 337409	[92]
	14 varieties	*A. parasiticus*	Fungal sporulation recorded	Higher moisture reduces infection rate during IVSC	J-11, Lampang	[93]
	12 breeding and germplasm lines	*A. flavus*, *A. parasiticus*	Immunoaffinity column fluorometer method	Highly significant (E), (G) and (G × E) interactions identified	AR-2, GFA-1	[94]
**IVSC and AP**	25 breeding lines and cultivars of Africa	*A. flavus* and *A. parasiticus*	ELISA for toxin estimation	VAR 27 variety produced least aflatoxin but showed higher IVSC	ICGV 87084, ICGV 87094, ICGV 87110	[12]
	67 CSL lines and varieties	*A. flavus*	Seed colonization test	Varieties with compact and thicker testa resistant to IVSC	12CS-104, 73-33	[16]
	850 cultivars and elite lines	*A. flavus*	Seed colonies and aflatoxin estimated	Some varieties are susceptible to IVSC but, resistant to AP	PI 337394F, PI337409 and UF71513	[95]
	561 germplasm lines	*A. flavus*	Seed infection percentage and aflatoxin production recorded	ICRISAT core collection has more resistance to IVSC than the China core collection	ICG 12625 (resistant to AP) and ICG 4750 (resistant to seed invasion)	[96]

IVSC: In vitro seed colonization; PAC: Pre-harvest aflatoxin contamination; AP: Aflatoxin production; PHAC: Post-harvest aflatoxin contamination; CSL: Chromosome Substitution Lines; E: Environment; G: Genotype; and G × E: Genotype × Environment.

**Table 2:** A summary of the transcriptome and proteome based discovery of key genes and pathways involved in aflatoxin contamination in groundnut.

Resistance Mechanism	Key Genes/TFs and Pathways Identified	Functional Description	References
**Aflatoxin Production (AP)**	*WRKY*	Stress regulative transcription factor	[32]
	Toll/Interleukin1 receptor-nucleotide binding site leucine-rich repeat (*TIR-NBS-LRR*)	Highly conserved disease resistant genes in plants	
	Ethylene responsive factors	Transcriptionally regulates jasmonate signaling pathway	
	Heat shock proteins	Regulates heat shock factors which play vital role in plant defense	
	Pathogenesis-related (PR) 1,2,5	Defense-related genes	[30]
	*NBS-LRR* genes	Disease resistance gene	
	*WRKY*	Stress regulative transcription factor	ICRISAT, Unpublished
	Ethylene responsive factors	Plays intermediary role in salicylic acid pathway	
	Linoleate 9S-lipoxygenase	Plays role in Jasmonic acid signal transduction pathway	
**Pre-Harvest Aflatoxin Contamination (PAC)**	*ABR1*	Ethylene responsive transcription factor and repressor of ABA signaling	[108]
	Pathogenesis related-2	Stress and defense responsive gene	
	*BIG*	Auxin transport gene	
	*WRINKLED1*	Controls fatty acid biosynthesis pathway	
	Defensin	Defense response	[109]
	*TIR*	Defense response	
	Chalcone isomerase 3	Flavonoids biosynthesis	
	*EM protein*	Stress response	
	Cupin/Oxalate oxidase	Seed storage protein	[97]
	Fatty acid desaturase 1	Regulates fatty acid-biosynthesis pathway	
	Lipoxygenase	Plays role in Jasmonic acid signal transduction pathway	
	Iso-Ara h3	Seed Storage protein	[98]
	LEA 4	Stress related protein	
	Cu/Zn superoxide dismutase II	Antioxidant defensive protein	
	Heat shock protein	Regulates heat shock factors which play vital role in plant defense	
**In Vitro Seed Colonization (IVSC)**	Linoleate 9S-lipoxygenase	Plays role in Jasmonic acid signaling transduction	[31]
	Resveratrol synthase	Biosynthesize stilbene type-phytoalexins	
	Chalcone synthase	Flavonoids biosynthesis	
	Defensins	Defense response	
	Chitinases	Modulates immune response	
**Post-Harvest Aflatoxin Contamination (PHAC)**	Heat shock protein 70	Maintains internal cell stability like folding-unfolding of proteins	[106]
	Heat shock protein 90	Cellular immunity, signal transduction	
	*NB-LRR*	PAMPs perception	[99]
	Hypersensitive induced response protein	Hypersensitive response	
	S-locus glycoprotein	Induction of defense	
	Cytochrome P450	Degradation of toxins	
	Alcohol dehydrogenase-1F	Detoxification	
	SAM dependent isoflavone 7-O-methyltransferase	Biosynthesis of phytoalexins	
	Seed linoleate	Lipid metabolism	ICRISAT, Unpublished
	Resveratrol synthase	Biosynthesis stilbene type-phytoalexins	
	ABA responsive genes	Regulates stress responsive genes	
